# Supplementary material for: Population-Based Analysis of the Immunoglobulin G Response to Different COVID-19 Vaccines in Brazil
Source: Vaccines (Basel). 2022 Dec 22;11(1):21. doi: 10.3390/vaccines11010021 (PMC9862407; doi:10.3390/vaccines11010021)
Supplement: Supplementary file 1 [file vaccines-11-00021-s001.zip › vaccines-2014754-supplementary.pdf]

Supplementary Materials

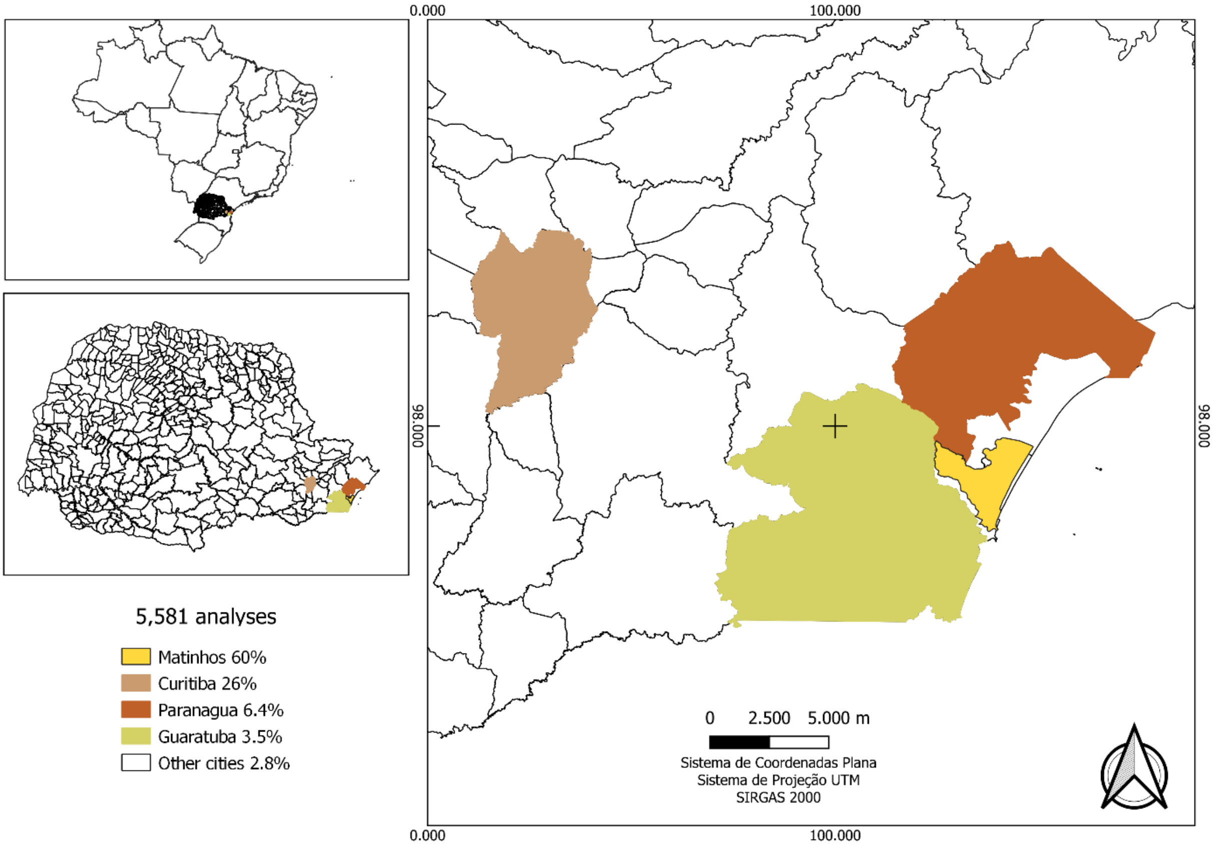

Figure S1: Location of the study site and city of residence of the participants

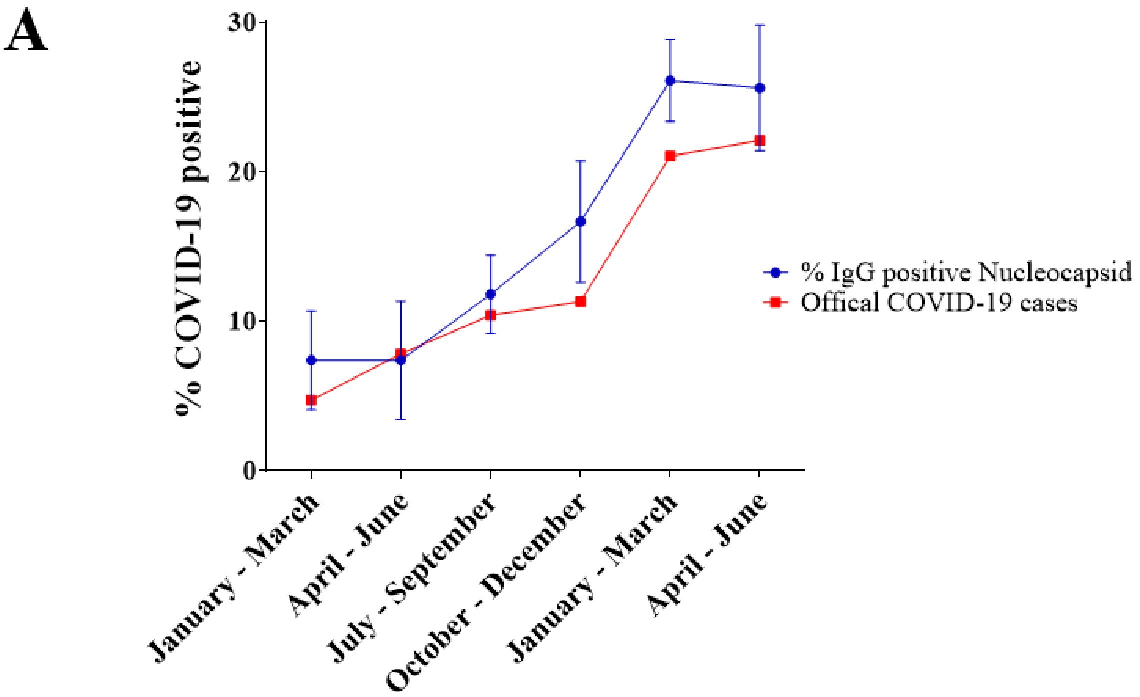

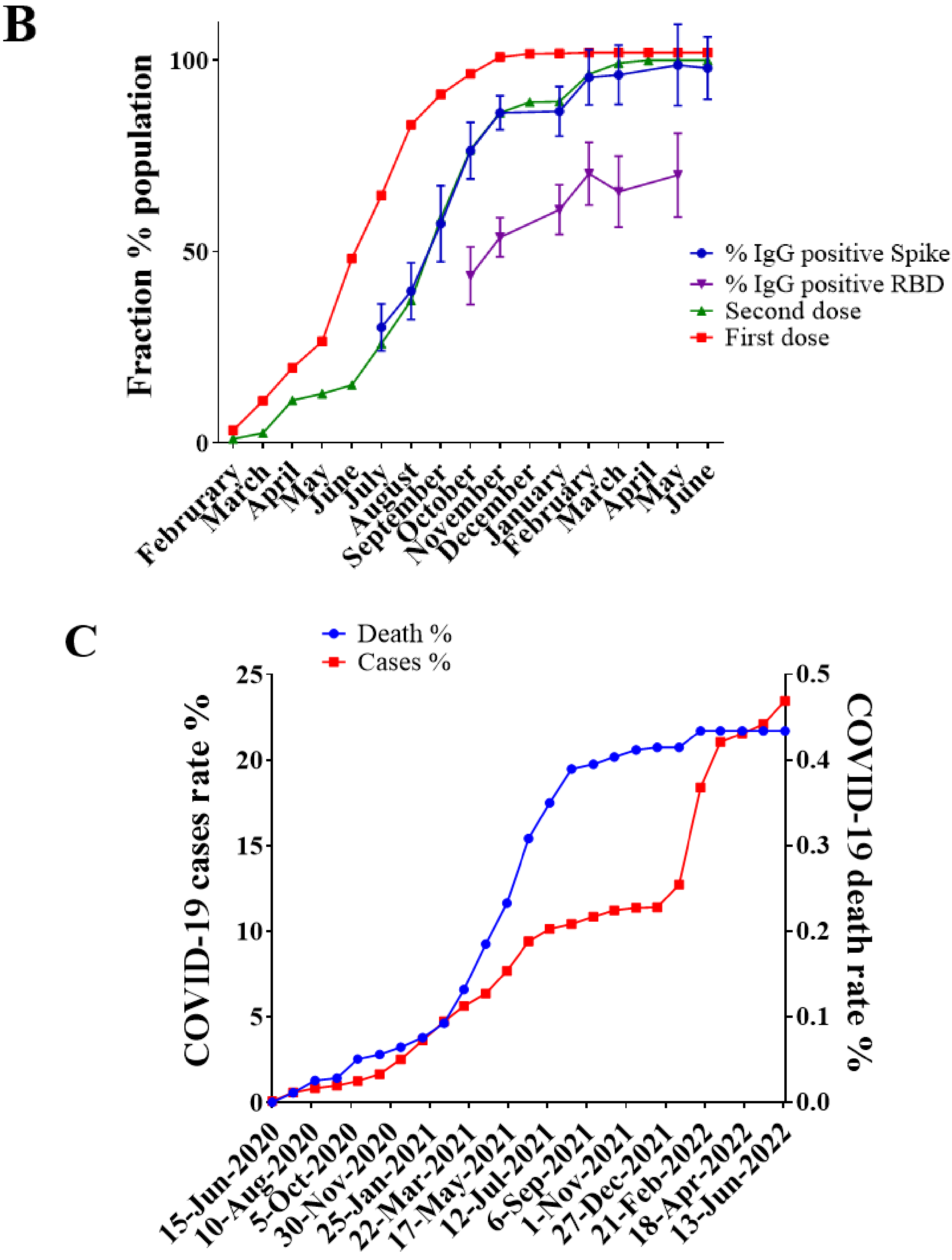

Figure. S2: Evolution of COVID-19 infection, vaccination and humoral response. (A) Evolution of COVID-19 during each quarter from January 2021 to June 2022. The percentage of samples IgG positive to the nucleocapsid antigen is represented by the blue line (error bars confidence levels at a 95% CI). The red line indicates the percentage of official cases of COVID-19 reported by health authorities in the city of Matinhos (cumulative cases in the middle of each quarter are reported as a percentage of the population). (B) The fraction of the population (>12 years) vaccinated with first dose (red), with the second dose (green) in the State of Paraná. The IgG seroconversion rate to Spike (blue) and RBD (purple). Error bars indicate confidence levels at a 95% CI. (C) Monthly evolution of official cases of COVID-19 (red) and death (blue) in the city of Matinhos.

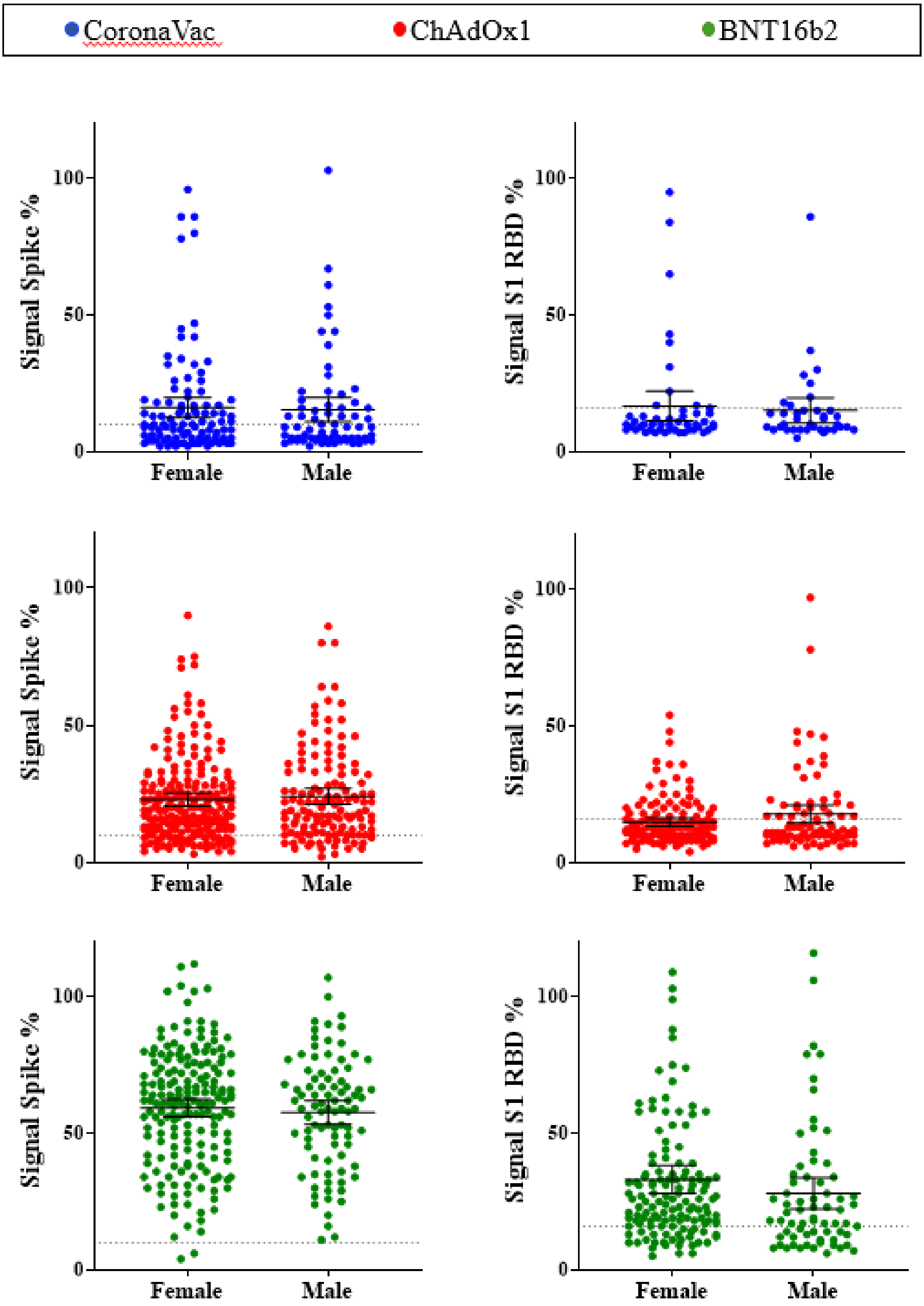

Figure S3: **IgG response to Spike and RBD according to gender**. IgG levels were determined in participants negative for COVID-19 which completed primary vaccination (2nd dose) between 10 to 240 days. The geometric mean is indicated with error bars reporting 95% CI. Gender was not statistically significant in all comparisons ($p>0.05$) in paired t-test.

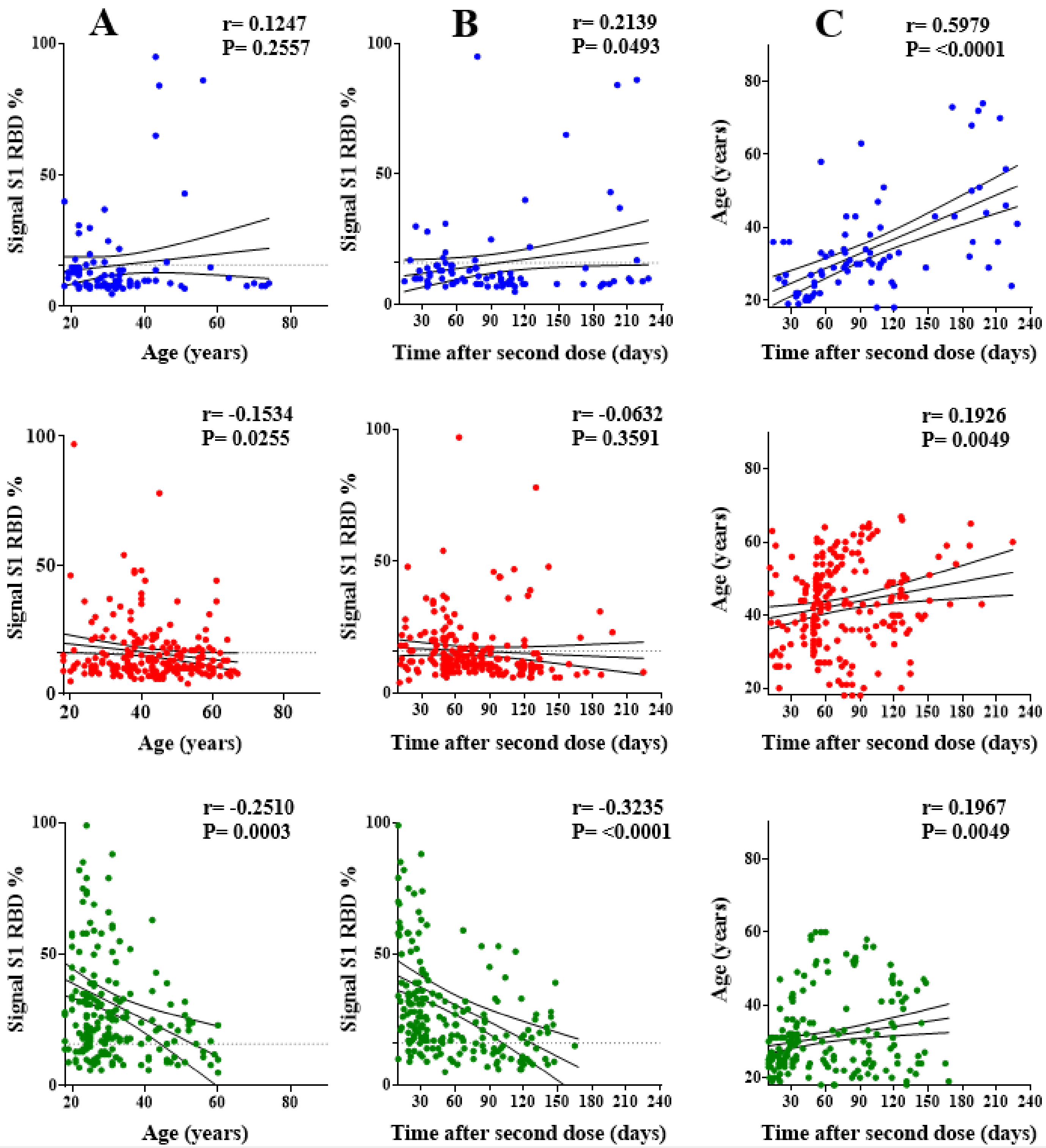

Figure S4: Correlation between the levels of IgG reactive to S1 RBD and the age or time after the second vaccine dose. The IgG levels were determined in participants negative for COVID-19 which completed primary vaccination (2nd dose) between 10 to 240 days. (A) Correlation between IgG levels and age. (B) Correlation between IgG levels and time after the second dose. (C) Correlation between Age and time after the second dose. The linear regression with 95% CI and Pearson's correlation coefficient is indicated. Color code, green BNT162b2, red ChAdOx1, blue CoronaVac..

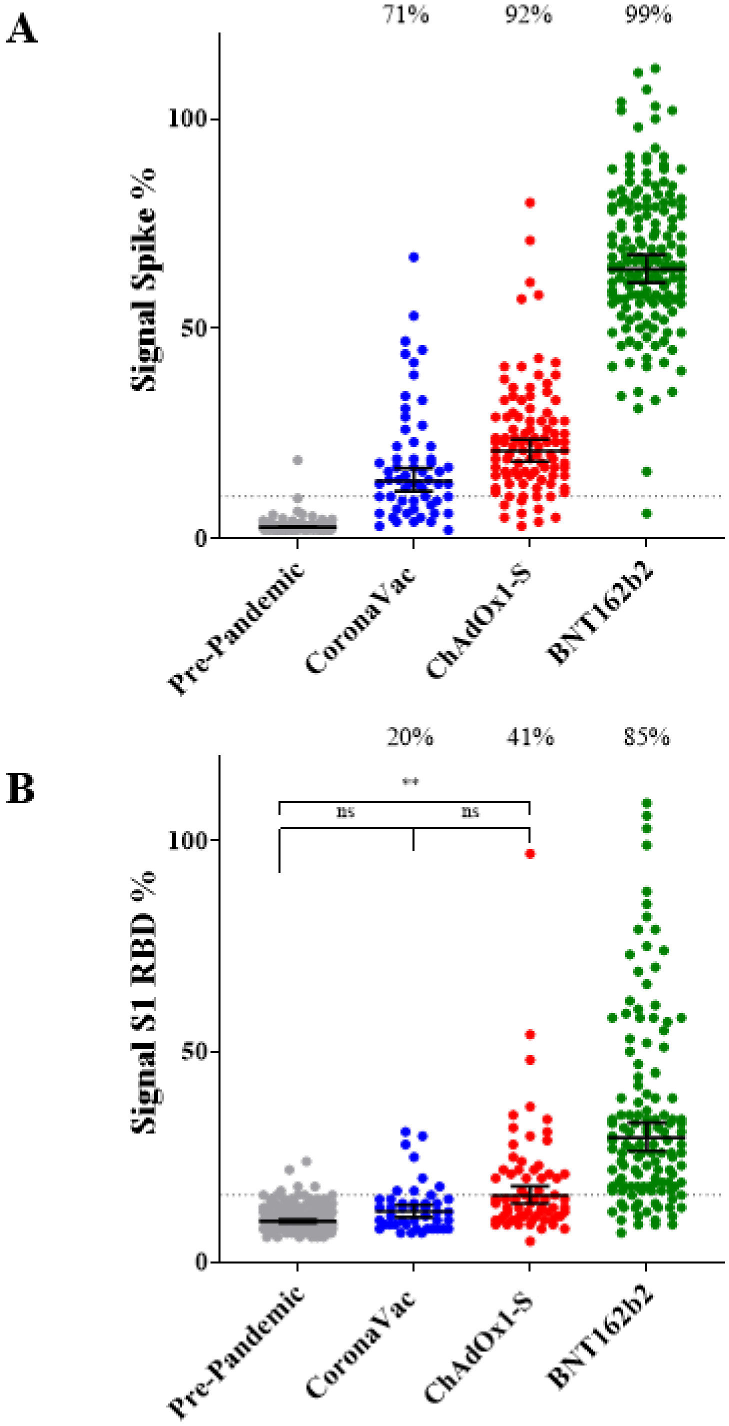

Figure S5: The IgG response to Spike and S1 RBD accordingly to the vaccine type. IgG levels reactive for Spike (A) and S1 RBD (B) in participants negative for COVID-19, with 18 to 40 years of age which completed primary vaccination (2nd dose) between 10 to 90 days. The dashed line indicates the seropositive assay cutoff. Pre-pandemic samples were plotted as naïve controls. The geometric mean and 95% CI is represented by the bars. One-Way ANOVA analysis were performed (**** p<0.0001), (**p<0.01), (ns, not significant). Number indicate the seroconversion rates.

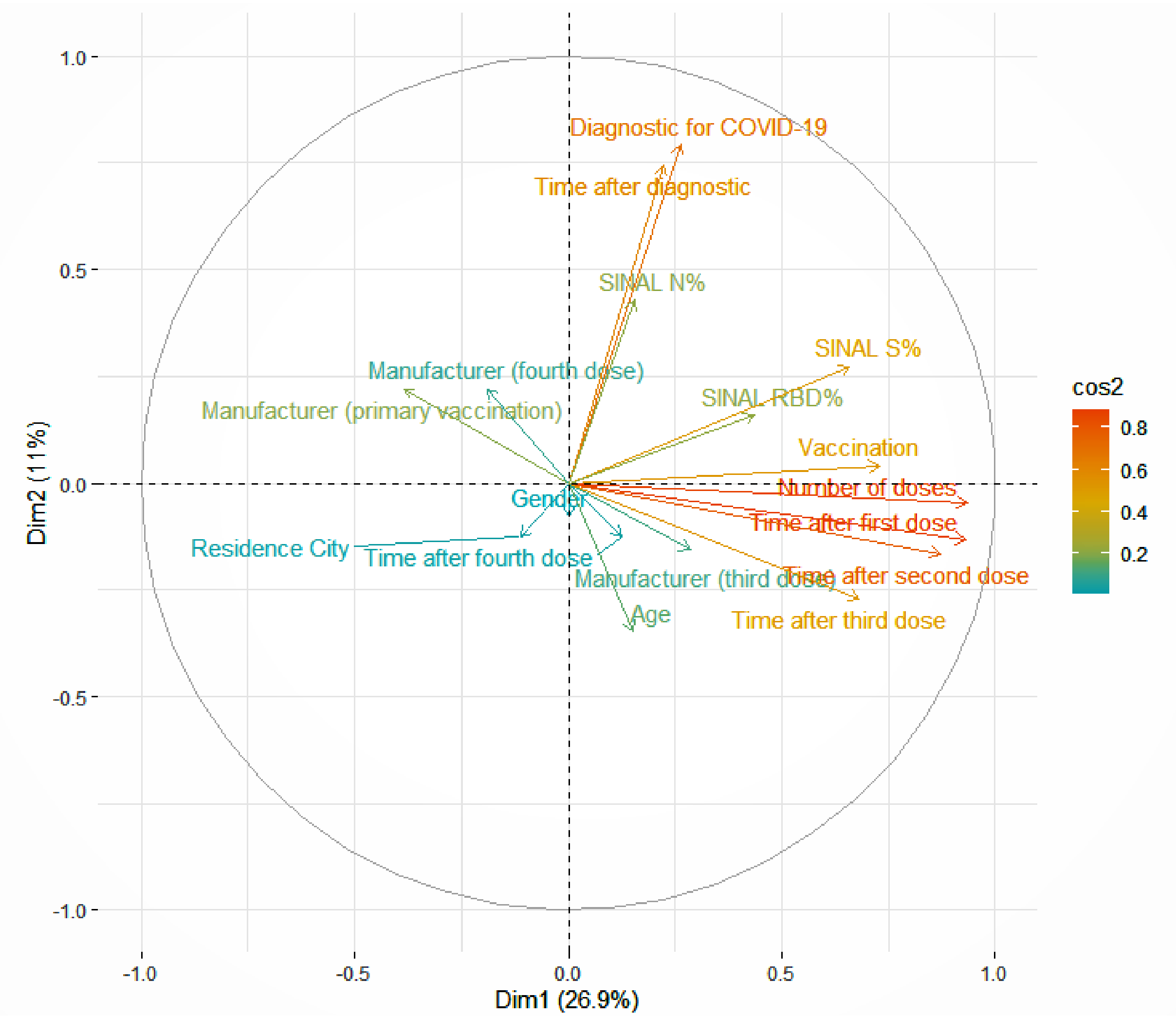

Figure S6 Principal Component Analysis (PCA). The table of variables was subject to PCA analysis. The influence of each variable in the variance of the dataset along PC1 and PC2 are indicated as vectors. Positively correlated vector variables are grouped together. The quality of representation of each variable for the variance of the dataset is indicated by the color gradient (strong to weak, red to blue)**.**
